# Towards an Implementation‐STakeholder Engagement Model (I‐STEM) for improving health and social care services

**DOI:** 10.1111/hex.13808

**Published:** 2023-07-04

**Authors:** Sebastian Potthoff, Tracy Finch, Leah Bührmann, Anne Etzelmüller, Claire R. van Genugten, Melissa Girling, Carl R. May, Neil Perkins, Christiaan Vis, Tim Rapley, ImpleMentAll consortium

**Affiliations:** ^1^ Department of Social Work, Education, and Community Wellbeing Northumbria University Newcastle Upon Tyne UK; ^2^ Department of Nursing, Midwifery and Health Northumbria University Newcastle upon Tyne UK; ^3^ Clinical, Neuro‐, & Developmental Psychology Faculty of Behavioural and Movement Sciences VU Amsterdam The Netherlands; ^4^ Department Sports and Health Sciences Technical University of Munich Munich Germany; ^5^ HelloBetter, GET.ON Institute für Online Gesundheitstrainings GmbH Hamburg/Berlin Germany; ^6^ Amsterdam Public Health Research Institute—Mental Health Amsterdam The Netherlands; ^7^ Department of Health Services Research and Policy London School of Hygiene and Tropical Medicine & NIHR North Thames ARC London UK; ^8^ Department of Public and Occupational Health Amsterdam Public Health Research Institute Amsterdam UMC The Netherlands; ^9^ Section for Research‐Based Innovation, Forhelse Research Centre for Digital Mental Health Services Division of Psychiatry Haukeland University Hospital Bergen Norway

**Keywords:** co‐design, co‐production, implementation strategies, normalisation, stakeholder engagement, tailored implementation

## Abstract

**Background:**

The implementation science literature acknowledges a need for engagement of key stakeholders when designing, delivering and evaluating implementation work. To date, the literature reports minimal or focused stakeholder engagement, where stakeholders are engaged in either barrier identification and/or barrier prioritisation. This paper begins to answer calls from the literature for the development of tools and guidance to support comprehensive stakeholder engagement in implementation research and practice. The paper describes the systematic development of the Implementation‐STakeholder Engagement Model (I‐STEM) in the context of an international, large‐scale empirical implementation study (ImpleMentAll) aimed at evaluating the effectiveness of a tailored implementation toolkit. The I‐STEM is a sensitising tool that defines key considerations and activities for undertaking stakeholder engagement activities across an implementation process.

**Methods:**

In‐depth, semistructured interviews and observations were conducted with implementers who were tailoring implementation strategies to integrate and embed internet‐based cognitive behavioural therapy (iCBT) services in 12 routine mental health care organisations in nine countries in Europe and Australia. The analytical process was informed by principles of first‐ and third‐generation Grounded Theory, including constant comparative method.

**Results:**

We conducted 55 interviews and observed 19 implementation‐related activities (e.g., team meetings and technical support calls). The final outcome of our analysis is expressed in an initial version of the I‐STEM, consisting of five interrelated concepts: engagement objectives, stakeholder mapping, engagement approaches, engagement qualities and engagement outcomes. Engagement objectives are goals that implementers plan to achieve by working with stakeholders in the implementation process. Stakeholder mapping involves identifying a range of organisations, groups or people who may be instrumental in achieving the engagement objectives. Engagement approaches define the type of work that is undertaken with stakeholders to achieve the engagement objectives. Engagement qualities define the logistics of the engagement approach. Lastly, every engagement activity may result in a range of engagement outcomes.

**Conclusion:**

The I‐STEM represents potential avenues for substantial stakeholder engagement activity across key phases of an implementation process. It provides a conceptual model for the planning, delivery, evaluation and reporting of stakeholder engagement activities. The I‐STEM is nonprescriptive and highlights the importance of a flexible, iterative approach to stakeholder engagement. It is developmental and will require application and validation across a range of implementation activities.

**Patient or Public Contribution:**

Patient contribution to ImpleMentAll trial was facilitated by GAMIAN‐Europe at all stages—from grant development to dissemination. GAMIAN‐Europe brings together a wide variety of patient representation organisations (local, regional and national) from almost all European countries. GAMIAN‐Europe was involved in pilot testing the ItFits‐toolkit and provided their views on the various aspects, including stakeholder engagement. Patients were also represented in the external advisory board providing support and advice on the design, conduct and interpretation of the wider project, including the development of the ItFits‐toolkit.

**Trial registration:**

ClinicalTrials.gov NCT03652883. Retrospectively registered on 29 August 2018.

## 1 INTRODUCTION

In implementation research, there is a growing recognition that effective implementation requires engagement of stakeholders in the design, delivery and evaluation of implementation strategies. A stakeholder can be defined as any ‘individual or group who is responsible for or affected by health‐and healthcare‐related decisions that can be informed by research evidence’.^1^ Comprehensive stakeholder engagement can lead to a better understanding of local needs and barriers, increased relevance and impact of implementation activities, improved implementation capacity and capability and increased research adoption.^2^ Beyond these instrumental benefits, there are also wider normative and political reasons for engaging stakeholders, including a shift from a paternalistic ‘science advice’ model to a more democratic and inclusive model of knowledge exchange.^3^

The wider literature provides a range of approaches to stakeholder engagement. Research on patient and public involvement (PPI) focuses on a subset of stakeholders and emphasises the need for shifting power towards the beneficiaries of services to co‐produce mutually valued outcomes.^4^, ^5^ Co‐creation and participatory action research go beyond the usual stakeholder engagement by emphasising the development and maintenance of bidirectional relationships.^6^ Implementation science can be informed by these literatures, to incorporate more comprehensive approaches to stakeholder engagement.

Stakeholder engagement is highly relevant for all stages of the implementation process (i.e., exploration, preparation, implementation and sustainment).^7^ Engaging stakeholders across the implementation process can facilitate the identification of context‐specific barriers and the matching of more acceptable implementation strategies. Most of the work to date often reports minimal stakeholder engagement in the implementation process.^8^, ^9^, ^10^, ^11^, ^12^, ^13^, ^14^, ^15^ Stakeholders are either not directly engaged in the implementation process (e.g., a core team, often the researchers, decide on the goals and strategies) or they are minimally engaged (e.g., through brief periods of formal or informal feedback). In other implementation work, stakeholders are formally engaged, generally through single methods, in discrete aspects of the process, either barrier identification or barrier prioritisation.^16^, ^17^, ^18^ None of the work reported to date formally engages stakeholders in a more comprehensive manner, in all aspects of the implementation process—from barrier identification, to barrier prioritisation, to implementation strategy selection, to intervention design.

Within the implementation literature there has been a call for the development and validation of tools and guidance to facilitate more comprehensive approaches to stakeholder engagement.^19^ Existing approaches to stakeholder engagement include guiding principles,^1^, ^20^ taxonomies,^21^ mapping criteria^22^, ^23^, ^24^, ^25^ and other classification systems.^26^, ^27^ For example, based on existing literature and empirical insights, Boaz et al.^20^ formulated ‘design principles’ to support stakeholder engagement in implementation (e.g., clarify objectives of stakeholder engagement). Similarly, Concannon and colleagues^1^ formulated a framework for classifying stakeholders in comparative effectiveness research, followed by recommendations for how to engage stakeholders. While guiding principles are helpful for designing stakeholder engagement activities, they do not offer enough conceptual depth to support the monitoring or evaluation of such activities.

Some approaches used in implementation focus on mapping and prioritising stakeholders using predefined criteria. For example, an interest–influence matrix is often used to determine the potential influence of stakeholder groups in a project.^25^ Others have used the criteria power, legitimacy and urgency to generate typologies of stakeholders and their importance to a project.^22^ One of the limitations of existing mapping criteria is that they were developed in management research, which focuses on the strategic aims of organisations rather than on the potential benefits of stakeholder engagement in implementation research.

Other approaches have focused on classifying different degrees, rather than on qualities, of stakeholder engagement. For example, the Spectrum of Public Participation classifies stakeholder engagement on a continuum including inform, consult, involve, collaborate and empower.^27^ Another classification system distinguishes nonparticipation, symbolic participation and engagement participation.^26^ While these classification systems begin to offer some conceptual clarity, they are still limited to certain aspects of stakeholder work and do not cover the range of considerations needed across an implementation process (e.g., setting objectives or evaluating outcomes).

Lastly, we identified two approaches that offer more comprehensive guidance for stakeholder engagement in implementation, including the ‘analytic‐deliberative’ model^28^ and the dynamic adaptation process (DAP) model.^29^ The analytic‐deliberative model was developed with contributions from qualitative and quantitative research (literature review, expert panel and pilot study) and offers a simple process for engaging stakeholders including inputs, methods, outputs and processes.^30^ Similarly, the DAP describes possible stakeholder engagement across the implementation process.^29^ In the DAP adaptations to an evidence‐based practice need to be planned and coordinated in close collaboration with an Implementation Resource Team (IRT) and other key stakeholders to preserve fidelity to core components.^29^ While these process models provide clear ‘how‐to’ guidance for stakeholder engagement, they do not provide much conceptual depth with regard to the different dimensions of stakeholder work across the implementation process.

In this article, we aim to advance current approaches to stakeholder engagement by describing the systematic development of an initial conceptual model for implementation‐stakeholder engagement. As part of a multinational, Horizon 2020‐funded study,^31^, ^32^ we conducted an in‐depth qualitative investigation of stakeholder engagement activities undertaken by implementation teams who used a toolkit designed to support the tailored implementation of digital mental health services across nine countries. We developed an initial Implementation‐STakeholder Engagement Model (I‐STEM) through our investigation of what happens when implementers are structured into iterative cycles of stakeholder engagement across various phases of the implementation process, but where they retain considerable flexibility in how they approach this engagement work. We define a ‘model’ as a coherent conceptual arrangement that, when it is operationalised, makes possible a rationale description and taxonomy of a phenomenon. We have developed the I‐STEM to support implementers with the planning, delivery and evaluation of stakeholder engagement activities when translating research into practice. The I‐STEM guides implementers through the different phases of a stakeholder engagement process and can be used flexibly alongside existing theories, models and frameworks of implementation.^33^

## 2 METHODS

### 2.1 Study design and settings

This study was conducted alongside the ImpleMentAll (IMA) effectiveness trial comparing a toolkit for tailored implementation (ItFits‐toolkit) with implementation as usual activities in a stepped‐wedge study design.^31^ Here we draw on the findings from a qualitative process evaluation, which was conducted alongside the effectiveness trial to understand how implementers engaged with the toolkit and how they worked with key stakeholders throughout the different phases of the implementation process. The study design consisted of qualitative interviews with members of the implementation teams and observations of meetings and events related to the implementation work. Members of the site implementation teams included: implementation leads (ILs; those responsible for coordinating the implementation work), core team members who were part of the teams working closely with ILs and other relevant stakeholders. The study settings included 13 implementation sites from nine counties—Italy, Spain, Germany, France, The Netherlands, Denmark, Kosovo, Albania and Australia. Even though each of the sites worked on implementing digital mental services, there was considerable variability with regard to the stages of implementation that sites were at. Some of the sites already had established regional expert centres and aimed to scale out their services, whereas other sites had only recently started their service and were still in the preparation phase. This variability provided us with a natural laboratory to explore how implementers worked with stakeholders across all stages of an implementation process. A detailed study protocol for the qualitative process evaluation can be found in Supporting Information: Additional File 1. A completed checklist of the ‘Standards for Reporting Qualitative Research’ can be found in Supporting Information: Additional File 2.^34^

### 2.2 ItFits‐toolkit approach to stakeholder engagement

The implementation toolkit tested in the IMA trial had a build‐it requirement for implementers to engage in iterative cycles of stakeholder engagement. The toolkit allowed considerable flexibility and did not specify how stakeholder engagement was to be undertaken. Instead, it provided implementers with a range of processes, principles and resources that they could use to enable stakeholder engagement activities, including:

1. *Core team*: A broad range of internal and external stakeholders who led and coordinated the implementation work locally using the ItFits‐toolkit.
2. *Core principles*: Six core principles (including ‘be open’) that highlight the importance of listening and valuing stakeholders' knowledge and experience.
3. *Module steps*: All four modules of the ItFits‐toolkit (identify, match, design and apply/review) incorporate three important substeps: (1) initialise; (2) verify and discuss and (3) finalise. First, the core team formulates the initial ideas in each of the modules (e.g., potential barriers to implementation). Next, they engage with a wider group of stakeholders to verify, discuss and potentially expand on those ideas. Lastly, the core team finalises each module (i.e., considering the inputs of all involved stakeholders).
4. *Consensus techniques*: Instructions on how to carry out brainstorming, structured group discussions, informal conversations, email discussions and surveys. An integrated online tool for creating custom surveys that can be sent out to stakeholders to collect information for tailoring implementation strategies.

### 2.3 Data collection

#### 2.3.1 Interviews

Interviews with ILs, core team members and stakeholders within implementation sites were conducted repeatedly throughout the trial period using a theory‐informed topic guide that evolved over time.^35^ Core team members and stakeholders were sampled and interviewed to gain in‐depth data on specific issues, and develop, test and then refine emerging analytic ideas. Interviews were mostly on a one‐to‐one basis and were conducted using video conferencing technology. All interviews were conducted in English by an experienced qualitative researcher (S. P.).

#### 2.3.2 Observations

Meetings and events related to implementation activities were observed, either in real time or via video recordings made by site and project team members. These included technical trial support calls, on‐demand technical support, follow‐up calls and core team meetings. All support activities were delivered by the central research team either face‐to‐face or via videoconferencing technology. A member of the process evaluation team (S. P.) was present in these activities as a nonparticipating observer.

### 2.4 Data collected

Overall, 55 interviews were conducted with 30 individual participants across the study duration. The number of interviews contributed by each site ranged between 2 and 7 (median = 4). Interviewees were ILs (*n* = 19), core team members (*n* = 9) and other stakeholders (*n* = 2). The number of ILs exceeds the number of sites due to staff turnover. Some interviewees took part in more than one interview. IL and core team member participants were professionals (both clinicians and support staff) working within healthcare within both private and public sectors who were involved in the implementation of digital mental health services. Other individuals included stakeholders who had key roles in relation to the work undertaken through the ItFits‐toolkit, but who were not considered part of the core implementation team, for example, key facilitators working in collaborating organisations that form part of the service delivery (e.g., insurance companies). In addition to interviews, we collected observational data during 19 calls: monthly support calls (*n* = 9); 1‐month follow‐up support calls (*n* = 5) and 3‐month follow‐up support calls (*n* = 5). The total number of sites participating in each of the recorded calls ranged from 1 to 5. The number of calls that sites took part in ranged from 1 to 4.

### 2.5 Data management and anonymisation

Participant written consent was taken for all data collection activities. Interviews were audio‐recorded and transcribed verbatim for analysis. Meetings and events were observed and fieldnotes were taken. All data were carefully anonymised to prevent identification of either the individual participant or the participating study site. Qualitative software (NVivo) was used to support data management, analysis and documentation.

### 2.6 Data coding and interpretation

**Figure 1:**
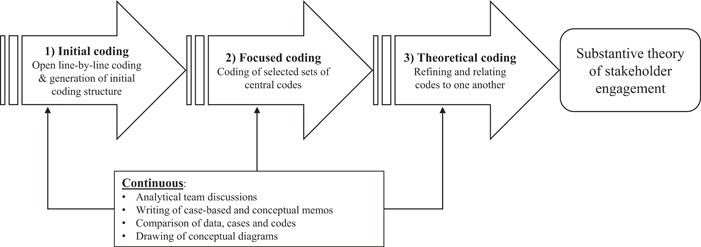
Analytical approach applying the principles of the constant comparative method.

The analytical process for the interviews and observations was informed by principles of first‐ and third‐generation Grounded Theory.^36^ Sampling, data collection and analysis were iterative. Data were analysed and categorised using the *constant comparative method* of data analysis,^37^, ^38^ involving initial line‐by‐line coding, focused coding, theoretical coding and the production of memos (Figure 1). The ongoing analysis informed further rounds of sampling and data collection as concepts started to emerge (*theoretical sampling*). The research team discussed and refined the emerging codes and categories throughout the analytical process. They created, reviewed and refined analytical memos, conceptual maps and diagrams, integrating concepts over time. The resulting *initial* model for implementation stakeholder engagement is the outcome of an iterative model development process, which was conducted alongside the qualitative data analysis. This process involved building numerous iterations of models through diagrams and applying those models to samples of qualitative data, seeking out both conflicting and confirming examples in relation to the models. After each round of model application, the team worked collectively to refine diagrams and concept definitions, until we arrived at the most parsimonious constellation of concepts that represented the analytic insights developed from the data. To illustrate the iterative development process, we have included two early versions of the I‐STEM in Supporting Information: Additional File 3.

## 3 RESULTS

### 3.1 I‐STEM

**Figure 2:**
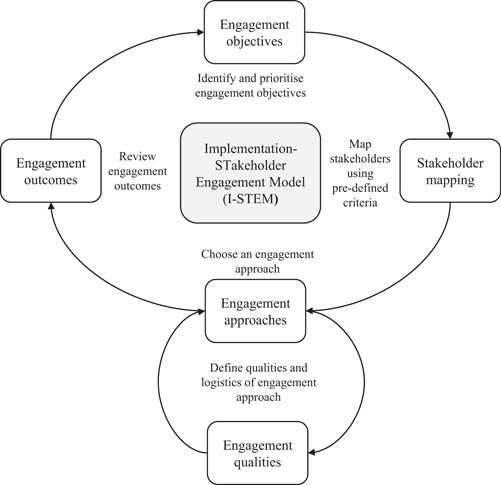
Implementation‐STakeholder Engagement Model (I‐STEM) for implementing evidence‐based practices. The I‐STEM is a sensitising tool that defines key considerations and activities for undertaking stakeholder engagement activities across an implementation process.

We have developed the I‐STEM (Figure 2) showing how stakeholders can be formally engaged in the implementation process. I‐STEM builds on the idea that comprehensive engagement of stakeholders in all aspects of the implementation process can help (re)shape the work in constructive ways, increase ownership of the process and subsequent uptake of the innovation. The I‐STEM is not prescriptive but rather highlights the importance of a flexible, iterative approach to stakeholder engagement. It is a sensitising tool that can be used alongside existing implementation theories, models and frameworks to support the planning, delivery and evaluation of stakeholder engagement activities by focusing on four key processes:

- Identify and prioritise engagement objectives.
- Map stakeholders using pre‐defined criteria.
- Choose an engagement approach.
  - And define qualities and logistics of the engagement approach.
- Review engagement outcomes.

We will now outline the core processes of I‐STEM, using examples from work in IMA, to demonstrate key points. In Supporting Information: Additional File 4, we provide an applied case study example from one of the IMA trial sites that demonstrates the workability of the I‐STEM.

### 3.2 Engagement objectives: Identify and prioritise engagement objectives

Engagement objectives are the goals that implementers are hoping to achieve by working with stakeholders in the implementation process. Clear objective setting, whether set before initiating any engagement activity or co‐developed with stakeholders, is key as it asks people to actively reflect on and be explicit about the scope and the type of activity to be carried out. Implementers can decide to engage with stakeholders to achieve a range of key objectives (see Table 1 for full descriptions). These objectives might include plans to:

- Inform
- Understand
- Verify
- Enrol
- Access
- Do

**Table 1:** Possible objectives for engaging stakeholders in implementation work.

Engagement objectives		
Focus of engagement work	Description	Illustrative example
Inform	The objective of engagement activities may focus on serving to inform and raise stakeholders' awareness of the innovation.	*Informing* involved telling practitioners ‘what the service offers to the users’ (Site 1, interview 3) as well as *informing* patients ‘how to register’ (Site 1, interview 3) for the iCBT service. *Informing* was usually the objective where engagement activities aimed to increase stakeholders' awareness, including awareness about the evidence of effectiveness, awareness about which patients might benefit and which likely not, or of awareness about payment and reimbursement structure.
Understand	The objective of engagement activities may focus on impacting on implementers' understanding of implementation‐related issues.	The ItFits core principle ‘be open’ encouraged implementers to value stakeholders' views and experiences. Consequently, many of the implementers reported reaching out to stakeholders to *understand* their views ‘on their difficulties or what already works good’ (Site 1, interview 1). Implementers reached out to stakeholders at various points throughout the tailoring process to *understand* their views on goals, barriers and strategies related to the upscaling of iCBT services.
Verify	The objective of engagement activities may focus on verifying implementers' initial ideas about the implementation activities.	Work in the ItFits‐toolkit involved the core implementation team *verifying* their initial ideas with stakeholders. Implementers reported *verifying* their initial ideas using various consensus techniques provided in the toolkit (e.g., brainstorming, interviews and informal conversations). For example, implementers brainstormed determinants of practice and subsequently asked stakeholders to complete a questionnaire to *verify* and prioritise their initial ideas.
Enrol	The objective of engagement activities may focus on serving to enrol stakeholders in the implementation process.	A common engagement objective across sites was to *enrol* stakeholders in the implementation process. *Enrolling* included both gaining initial buy‐in from stakeholders as well as maintaining trust throughout the implementation process. Consistent staff turnover meant that *enrolling* stakeholders was often an ongoing process involving ‘meeting new colleagues and having to explain again this is what we're doing’ (Site 2, interview 3).
Access	The objective of engagement activities may focus on impacting on financial or material resources available for the implementation work.	A common objective was to *access* additional financial or material resources for the implementation of iCBT services, or specialist expertise (e.g., IT). Some sites were completely reliant on project funding to keep their services running, so they reached out to commissioners to secure *access* to future funding. One site hired a new marketing team to gain *access* to new service users and overcome low uptake rates.
Do	The objective of engagement activities may focus on practically doing elements of the implementation work.	Stakeholder engagement served to practically *do* the implementation work. For example, in one site the core implementation team developed an implementation blueprint in which ‘different people are responsible for doing these different steps, so they are like the experts of their step’ (Site 1, interview 1). In another site flyers and posters were disseminated in practice waiting rooms to get people to undertake screening for depression, which people could do on their own devices.

Abbreviations: iCBT, internet‐based cognitive behavioural therapy; IT, information technology.

Importantly, objectives may change throughout the engagement activity and each activity may address one or more objective(s). For example, implementers may want to *enrol* stakeholders in an implementation activity by *informing* them about the specifications and advantages of an innovation. Subsequently, implementers may want to *understand* stakeholders' views regarding barriers and facilitators to the implementation of the innovation. Or implementers may already have some initial ideas about barriers and facilitators (e.g., from reviewing the literature) and want to *verify* these ideas with the stakeholders.

### 3.3 Stakeholder mapping: Map stakeholders using pre‐defined criteria

Once implementation objectives have been formulated it is crucial to identify, or map, a range of stakeholders who may help to achieve them. Implementers need to consider why they want to engage with specific stakeholder organisations, groups and individuals. Not all the potential reasons for engaging with stakeholder will be relevant for every engagement activity (see Table 2 for full descriptions), so implementers may choose to focus on selecting reasons from the following criteria:

- Influence
- Expertise
- Orientation
- Impact
- Capacity
- Trust

**Table 2:** Possible reasons for choosing stakeholders for implementation activities.

Stakeholder mapping		
Reason for working with specific stakeholders	Description	Illustrative example
Influence	The degree to which stakeholders have influence on the implementation of an innovation, either directly or via other stakeholders.	Implementers reported selecting stakeholders because of their *influence* on the implementation of iCBT services, either directly or via other stakeholders. For example, in one site, implementation core team members were selected to include executive representatives from each of the implementation sites to guarantee optimal support for their activities. In another site implementers reflected that *not considering* a group of *influential* stakeholders at the outset, delayed their project, as their sign off on implementation activities was required.
Expertise	The degree to which stakeholders have information, counsel or expertise relevant to the implementation of the innovation.	Stakeholders *expertise*, information or counsel relevant to the implementation of the iCBT service was considered. For example, implementers in one site acknowledged that they chose stakeholders ‘who are most likely the ones who can do that task best’ (Site 3, interview 4). This included an IT expert who ‘knows about web pages’ (Site 3, interview 4) and a journalist ‘who can do communication’ (Site 3, interview 4). Clinical experience relevant to the implementation activity was also considered.
Orientation	The degree to which stakeholders' views or attitudes towards the innovation are favourable or resistant.	Stakeholders were selected because of their *orientation* (views or attitudes) towards the iCBT service. Orientation was perceived to be related to stakeholders' willingness to participate in the engagement activity. For example, in one site pharmacists were chosen because they were seen as ‘highly engaged and interested in the project because they see the value in eHealth’ (Site 4, interview 5). In another site, a stakeholder was selected because they were a known sceptic of the innovation and likely to contribute useful perspectives as devil's advocates.
Impact	The degree in which the implementation project impacts on the stakeholder.	The potential *impact* on those delivering (directly or indirectly) or receiving the iCBT was used as another selection criterion. In one site, implementers decided to work with practitioners because of the likely *impact* that the implementation strategy (i.e., change to patient intake procedures) would have on their work. Similarly, many sites worked with patients because of the *impact* on the care that they would receive.
Capacity	The degree to which stakeholders have the capacity to take part in the engagement activity.	For example, some implementers selected stakeholders because they had additional time allocated for implementation activities and were likely to meet the commitments required for the engagement. In one site implementers avoided working with GPs because they had ‘extremely busy schedules’ (Site 4, interview 5), so instead they focused on pharmacists because they were more available and interested in the iCBT service.
Trust	The degree to which there is mutual familiarity and trust between the potential stakeholders.	Mutual familiarity and *trust* were considered important when selecting stakeholders. Often this involved working with stakeholders who were known to adhere to commitments, respecting confidentiality and engaging in a productive dialogue. In one site a particular stakeholder group was avoided because they were thought to potentially interfere with the implementation process.

Abbreviations: iCBT, internet‐based cognitive behavioural therapy; IT, information technology.

The relevance of different stakeholders to achieving specific objectives is likely to change over time as the implementation activities evolve and as stakeholders themselves make decisions, shift focus or change their opinions. For example, when planning a new implementation activity, implementers may want to bring together a range of internal and external stakeholders to co‐produce the implementation strategy. To ensure optimal uptake, implementers may decide to engage directors of the organisation because of their *influence* and understanding of the wider organisational context. At the same time, they may want to engage practitioners because of their *expertise* in delivering the innovation. Finally, they may engage people with lived experience because of the *impact* that the implementation activities will have on the services that they will receive.

### 3.4 Engagement approaches: Choose an engagement approach

The range of stakeholder engagement activities can be categorised according to the type of work that it involves. Four core ways of working with stakeholder may be considered by implementers (see Table 3 for full descriptions):

- Disseminating
- Assessing
- Consulting
- Collaborating

**Table 3:** Possible ways of working with stakeholders when implementing change.

Engagement approaches		
Ways of working with stakeholders	Description	Illustrative example
Disseminating	Disseminating involves giving out information about the innovation.	*Disseminating* information about the iCBT service was a common activity, and implementers reported using a range of *dissemination* strategies including multimedia campaigns, educational outreach visits or conferences. In one of the sites implementers *disseminated* information to practitioners, so that they knew ‘how they can be in touch with community mental health centres’ (Site 5, interview 2).
Assessing	Assessing involves gathering information from stakeholders that is relevant to the implementation activity.	A lot of the reported engagement activities involved *assessing* stakeholders’ views and experiences on an implementation activity. For example, in one of the sites implementers used a survey before and after educational lectures for healthcare professionals to ‘see how they benefit from this lecture’ (Site 6, interview 2). An assessment may be carried out at any stage of the implementation process, for example, during the development of an implementation intervention or during the assessment of the intervention's impact.
Consulting	Consulting involves offering implementation‐related information to selected stakeholders to seek their feedback or advice.	Much of the tailoring work involved *consulting* with stakeholders for feedback or advice on an implementation activity. Implementers working with the ItFits‐toolkit regularly consulted with people with lived experiences to understand the potential impact the implementation strategy would have on their care. In one site implementers consulted with practitioners to ‘prepare as good as possible materials for mental health professionals’ (Site 7, interview 2).
Collaborating	Collaborating involves working closely with stakeholders on a common objective relating to implementation.	Finally, many of the reported activities involved *collaborating* closely with stakeholders on a common objective relating to implementation. In one of the sites this involved ‘brainstorming meetings with the participants to develop the content of those [educational] documents’ (Site 8, interview 3). One implementer highlighted how ‘relationships are key and that relationship is being built on that collaborative approach to any activity we do’ (Site 9, interview 2).

Abbreviation: iCBT, internet‐based cognitive behavioural therapy.

Which engagement approach is most suitable depends on the engagement objective, the stage in the implementation process and on the time and resources available. Ethical and political commitments can also shape the choice of approach. Furthermore, it is important to consider the stakeholders' time and capacity when deciding on a specific engagement approach. For example, in one organisation, implementers may want to inform stakeholders about the rollout of an innovation. To achieve this goal, they may want to *disseminate* information (e.g., via a newsletter) to practitioners because of the impact that the implementation will have on their work. In another organisation, implementers may want to enrol people with lived experience in the implementation process. To achieve this goal, they may choose to *collaborate* with these stakeholders by involving them in all stages of the implementation process.

### 3.5 Engagement qualities: Define qualities and logistics of engagement approach

Regardless of the approach taken—be that through disseminating, assessing, consulting or collaborating—the qualities embedded in the stakeholder engagement work can vary. Engagement qualities refer to the ways in which engagement activities are planned and carried out. Implementers may want to define elements of the pragmatics of their engagement approach using one or several of the listed qualities below, which should be viewed on a continuum (see Table 4 for full descriptions):

- Preparedness
- Structure
- Activity
- Regularity
- Accountability

**Table 4:** Possible qualities of stakeholder engagement work.

Engagement qualities		
Qualities of stakeholder engagement work	Description	Illustrative example
Preparedness	Engagement can vary in terms of the degree to which stakeholders are prepared for the activity.	Engagement activities reported by the implementers varied in terms of the degree of *preparedness*. The ItFits core principle ‘be organised’ encourages implementers to think through implementation activities and capture ideas about implementation before engaging stakeholders. Across sites there was a high level of preparedness with one group of implementers regularly preparing and sharing ‘a draft [of ItFits worksheets] for the meeting’ with stakeholders. During the meeting implementers adapted the draft with the stakeholders and found this to be ‘very efficient’ (Site 8, interview 2).
Structure	Engagement can vary in the degree to which the activity is structured.	Implementers varied in their preference for *structure* in the approach to engagement. Many implementers highlighted the value of having a structure or a set of instructions to guide engagement activities. For example, structuring activities around identifying and matching barriers and facilitators to implementation made it ‘very clear what the discussion should be about and in our case that's really helpful’ (Site 10, interview 3). Other times implementers preferred a less *structured* approach, for example when meeting informally with stakeholders to understand their perspectives.
Activity	Engagement can vary in terms of how actively stakeholders are engaged in the implementation work.	The degree of *activity* in engagement activities varied. Many implementers reported engaging *actively* with stakeholders when, for example, co‐designing educational materials to facilitate the uptake of iCBT services. Other engagement activities required less active engagement, for example, where information was disseminated to stakeholders via multimedia campaigns.
Regularity	Some stakeholder engagement activities are carried out on a regular basis, whereas other activities are more sporadic.	Engagement activities reported by implementers varied in terms of the *regularity* with which they were carried out. For example, one site organised ‘weekly meetings with the core team’ (Site 2, interview 4) to progress through the ItFits‐toolkit and tailor implementation strategies in collaboration with key stakeholders. At other sites stakeholder engagement involved a one‐off workshop to design informational materials.
Accountability	Engagement may vary in terms of the degree to which stakeholders are hold accountable for their contribution.	Engagement activities varied in terms of the degree to which implementers and stakeholders were held *accountable* for their tasks and responsibilities. For example, in one site implementers ‘were able to divide the different tasks in the toolkit’ (Site 1, interview 3) among different stakeholders, which made it clear who in the team was *accountable* for a specific task.

Abbreviation: iCBT, internet‐based cognitive behavioural therapy.

The choice of quality needs to be fit for purpose, depending on the type of engagement approach chosen. For example, implementers may want to enrol practitioners in the implementation process by collaborating with them in the design of the implementation strategy. This could involve co‐design workshops with a high degree of *preparedness* and *structure*. Stakeholders could be prepared by sending them workshop materials and agendas ahead of time. During the workshops, a trained facilitator could lead stakeholders through several structured activities to co‐design the implementation strategy. Or implementers may want to consult the internal information technology (IT) team to access their expertise and help with the technical implementation of an innovation. This may involve *regular* meetings with the IT team during which specific tasks are defined and people are made responsible for their completion (high level of *accountability*).

### 3.6 Engagement outcomes: Review engagement outcomes

*Engagement outcomes* are linked to the *engagement objectives* set earlier in the process of I–STEM. Some outcomes can be classed as ‘soft’ in that they relate to stakeholders' and implementers' engagement with and perceptions of the implementation/engagement process. Other outcomes can be classed as ‘hard’ as they relate to objective or material outcomes. Implementers working with stakeholders to tailor implementation strategies can focus on six different outcomes (see Table 5 for full descriptions):

- Informing
- Understanding
- Verifying
- Enrolling
- Accessing
- Doing

**Table 5:** Possible outcomes of stakeholder engagement work.

Engagement outcomes		
Outcomes of engagement work	Description	Illustrative example
Informing	Engagement activities may result in stakeholders being more informed about the innovation that is being implemented.	Implementers reported that engaging with stakeholders increased their knowledge and awareness about how to implement iCBT services in their routine practice. For example, one site worked with stakeholders at a call centre to *inform* them about referral options for their iCBT service. After the delivery of a tailored educational programme, stakeholders felt more *informed*, but referral rates were still lacking behind, which led to further inquiries into barriers to referral.
Understanding	Engagement activities may result in providing a better understanding of stakeholders' needs, barriers and facilitators with regard to the innovation.	Engagement activities helped implementers get a better *understanding* of stakeholders' goals, barriers and facilitators with regard to implementing iCBT services. For example, one of the sites used electronic tablets in GP practices to screen people who might be eligible for receiving iCBT services. Although many patients screened positively, only a few signed up for iCBT. After including a question about reasons for not signing up for the service, implementers learned that patients thought the sign‐up process was too time‐consuming.
Verifying	Stakeholder engagement may serve to verify initial ideas relating to the implementation of an innovation.	The ItFits‐toolkit helped implementers with *verifying* their initial ideas with key stakeholders. Implementers thought that *verifying* their initial ideas with stakeholders ‘made sense and worked’ (Site 2, interview 4). While adding value to the tailoring process, *verifying* was also perceived as time‐consuming and different from implementers' usual ways of working. However, especially in the early stages of implementation, the benefits of *verifying* were perceived to outweigh the costs.
Enrolling	Engagement activities may result in a desired outcome by enrolling relevant people in the process.	Implementers reported that engagement activities helped in *enrolling* stakeholders in the tailoring process. For example, one of the sites organised monthly meetings to ‘motivate some therapists that have not really included some patients and give new information concerning the study’ (Site 8, interview 2). These monthly meetings were further used to co‐produce educational materials. They developed with them as it enabled everyone to ‘have in mind all the troubles and problems they [therapists] have been facing’ (Site 8, interview 2). This co‐production helped *enrol* therapists because it made them take ownership of the developed strategy.
Accessing	Stakeholder engagement may serve to access resources or expertise necessary for implementation.	Engagement activities helped the implementer with *accessing* the resources and the expertise necessary for implementing iCBT services. For example, one site reached out to other iCBT providers to access their expertise in cooperating with software developers to design and deliver iCBT solutions. Collaboration with other providers helped implementers access diverse perspectives, which informed their own plans of working with software developers.
Doing	Stakeholder engagement may serve to practically undertake elements of the implementation work.	Some of the implementers working with the ItFits‐toolkit reported that stakeholder engagement activities helped them with *doing* the implementation work. For example, one of the sites conducted educational outreach visits and sought collaborations with psychologists from different community mental health centres. This activity helped reduce providers' resistance to the iCBT service and facilitated the referral process.

Abbreviation: iCBT, internet‐based cognitive behavioural therapy.

Importantly, any engagement activity may result in several different outcomes. Regular consultation meetings with directors of an organisation may provide implementers with a better *understanding* of the organisational context, while also providing increased *access* to organisational resources to carry out the implementation activity. Structured co‐design workshops with patients throughout the implementation process may help to *enrol* them and practically *do* elements of the tailored implementation activity.

### 3.7 Using the I‐STEM in research and practice

We have developed the I‐STEM as a flexible tool that can be used by implementation researchers and practitioners to facilitate the planning and evaluation of stakeholder engagement activities. The I‐STEM is a sensitising tool that defines key considerations and activities for undertaking stakeholder engagement activities across an implementation process. Implementers could use the tables provided in the manuscript to help them design a blueprint for meaningful stakeholder engagement activities. Using the tables, implementers could work through each of the concepts of the model to help them reflect on the objectives of the engagement activities, the types of stakeholders they want to engage, how they want to engage them and what they want to achieve. After generating an initial blueprint, which highlights the risks and benefits of engaging stakeholders, implementers can use it to monitor and adapt their engagement activities as they progress through a project. The I‐STEM could also be used as an analytical framework or coding manual to help implementers make sense of stakeholder engagement work in implementation research. Such applications could enable the refinement and extension of the identified concepts into what could become a more formalised theory in the future.

## 4 DISCUSSION

We have developed an initial model for engaging stakeholders throughout all phases of the implementation process. The I‐STEM is the outcome of observing how a range of different implementation teams worked with stakeholders in a large international implementation trial. This work begins to offer some answers to calls from the literature for the development of practice‐based tools and guidance to facilitate more comprehensive approaches to stakeholder engagement in implementation science.^19^, ^20^, ^21^ Engaging stakeholders in the development, delivery and evaluation of implementation strategies brings many potential opportunities. It can facilitate the identification of context‐specific barriers to implementation and the matching of more acceptable and feasible strategies to promote evidence uptake. Active engagement of stakeholders throughout all phases of an implementation process can also facilitate a move away from ‘top down’ approaches to implementation and help democratise evidence use.

The I‐STEM builds on and extends current theorising around stakeholder engagement.^1^, ^28^ For example, I‐STEM emphasises the importance of jointly identifying and prioritising engagement objectives and desired engagement outcomes as a starting point for any stakeholder engagement activities. Being clear and transparent about intended objectives and desired outcomes can help build trust and facilitate buy‐in from all involved stakeholders. This may also help prevent potential challenges that may arise when working collaboratively with people from different professions and backgrounds (e.g., administrative burden, interpersonal conflict or costs to research).^39^

The I‐STEM suggests six criteria that implementers can use for mapping stakeholders to specific engagement objectives (influence, expertise, orientation, impact, capacity and trust). In the wider literature, there are various models and methods for identifying stakeholders, including that of Mitchell et al.,^22^ with their three criteria (power, legitimacy and urgency) to determine stakeholder significance. Scholes and Clutterbuck^23^ also use three criteria (influence, impact and alignment) to assess stakeholder groups, as did Murray‐Webster and Simon^24^ (power, interest and attitude), resulting in the identification of eight types of stakeholders and suggestions on how each should be managed. Most models used for stakeholder mapping originate from organisational studies and focus on effective ‘management’ of stakeholders. Our criteria were derived from extensive stakeholder work in implementation practice and focus on identifying and engaging, rather than managing, stakeholders who are key in identifying barriers and matching solutions to support implementation processes.

One of the key steps within the I‐STEM involves choosing an engagement approach designed to enable the attainment of engagement objectives. The four included engagement approaches (disseminating, assessing, consulting and collaborating) overlap with mechanisms defined in public engagement research. In this literature, three categories of engagement are often distinguished: communication, involving a one‐way flow of information from sponsors to stakeholders; consultation, in which information is conveyed from the stakeholders to the sponsor; and participation, in which information is exchanged between sponsors and stakeholders.^40^ While there are some overlaps, the I‐STEM adds the category of assessing, which involves understanding stakeholder perspectives. Furthermore, I‐STEM highlights that defining the engagement approach is not sufficient and that we also need to pay attention to the qualities with which the approach is carried out (level of preparedness, structure, activity, regularity and accountability). Defining these qualities is key given that many implementation activities are often characterised by sporadic, trial‐and‐error approaches to stakeholder engagement. For example, we found that many implementation teams we observed valued scheduling regular meetings with stakeholders and assigning tasks that held individuals accountable.

Outside the implementation science literature, there has already been an emergence of different models, theories and frameworks for engaging with stakeholders in health and social care research. The analytic‐deliberative model provides a framework for making explicit links between stakeholder inputs and deliberative methods to generate outputs relevant to comparative effectiveness research.^28^ Inputs include different types of evidence, including both research and practitioner, patient and consumer knowledge and experiences. Both quantitative and qualitative methods are used to generate outputs (e.g., useful evidence, efficient use of resources and improved health outcomes). While a simple linear input–output model sounds appealing, it may not apply easily to the complexities of everyday implementation work. Implementation often happens within a wider political and organisational context and therefore requires flexibility and responsiveness. The I‐STEM enables a flexible and iterative approach whereby engagement activities are revisited and adapted when necessary. In the IMA study, which I‐STEM emerged from, the need for an iterative approach became clear during the coronavirus disease 2019 outbreak. The quickly evolving situation meant that implementers had to stay flexible and adapt their ways of engaging with stakeholders (e.g., remotely using video conferencing technology).

The I‐STEM is firmly rooted within theories of implementation. In the IMA trial, the implementation work was directed by the ItFits‐toolkit, which was informed by Normalisation Process Theory (NPT). NPT explains the work of implementation, embedding and integration with a focus on the contributions of stakeholders, who work together to undertake the day‐to‐day work.^41^ The four key generative mechanisms of NPT are that the implementation activity makes sense to those involved or affected (coherence), can be engaged with as required (cognitive participation), can be achieved through working together (collective action) and can be appraised and reflected on to make improvements (reflexive monitoring). We suggest then that I‐STEM extends NPT further by providing direction on how these change mechanisms might be achieved—or strengthened—through strategic stakeholder engagement activity targeted towards improving the implementation process.

The model is also in line with the idea of contextual integration and tailored implementation.^42^ This work recognises that for an implementation strategy to be successful, it needs to be adapted and consider site‐, organisation‐ or group‐specific contextual issues. Another strength of the model is that it can be applied alongside existing theories, models and frameworks of implementation^33^ or other research‐informed tools to support implementation work. For example, it complements existing approaches to context analysis in implementation science and could be used to develop a stakeholder strategy in the Basel Approach for contextual ANAlysis (BANANA).^43^ Another implementation model that highlights the central role of stakeholder engagement is the Dynamic Adaptation Process (DAP) for adapting and sustaining evidence‐based practices.^29^ In the DAP adaptations to the evidence‐based practice need to be planned and coordinated in close collaboration with an Implementation Resource Team (IRT) and other key stakeholders to preserve fidelity to core components. The I‐STEM is consistent with this approach and can provide greater conceptual clarity with regard to how stakeholders can be engaged across the four phases of the implementation process (i.e., exploration, preparation, implementation and sustainment).

### 4.1 Limitations and future work

One limitation of this study is that it did not qualitatively or quantitively measure whether differences in stakeholder engagement accounted for improved implementation outcomes. Future research should focus on developing questionnaires that operationalise the dimensions of the I‐STEM to facilitate the quantitative validation of the model. The I‐STEM was developed in the context of a large international trial looking at the implementation of evidence‐based iCBT services for common mental health disorders. Although the study included various different services and implementation contexts, the focus on one particular setting (mental health) and service may limit the generalisability of the I‐STEM. Another limitation was that implementers engaged mostly with internal and external professional stakeholders, while patients, service users and citizens were less actively engaged. Future research is needed to test this initial model across a wider range of implementation contexts and types of stakeholders. More work is also needed to operationalise the I‐STEM and develop detailed ‘how‐to’ guidance and tools to support the use of the model in implementation work. Importantly, all future development work should be conducted in close collaboration with relevant stakeholders, including patient and public representatives.

## 5 CONCLUSION

Within the implementation science literature there has been an increasing recognition that individuals or groups in or affected by health‐ and healthcare‐related decisions, interventions or policies (called ‘stakeholders’) should have a say in the implementation process. The I‐STEM builds on the idea that implementation success may be increased by engaging closely and understanding the views, priorities and experiences of stakeholders, those who actually undertake and/or are impacted by the day‐to‐day work. Comprehensive stakeholder engagement is normatively desirable and has the potential to challenge our taken‐for‐granted ideas and practices. The I‐STEM begins to provide greater conceptual clarity with regard to how stakeholders can be engaged in a meaningful way throughout an implementation process. It is a flexible sensitising tool that can be used alongside existing theories, frameworks and models of implementation to support the planning and evaluation of stakeholder engagement activities and thereby support the implementation of evidence‐based care. The I‐STEM is developmental and will need to be tested and validated across a range of implementation contexts.

## AUTHOR CONTRIBUTIONS

Tracy Finch and Tim Rapley led on the qualitative process evaluation. Sebastian Potthoff conducted the interviews and observations. Sebastian Potthoff and Tim Rapley carried out the initial focused analysis in close collaboration with Tracy Finch, Melissa Girling, Neil Perkins and Carl R. May. Throughout the analyses, all co‐authors met regularly as a research team to discuss and refine the emerging stakeholder engagement model. Sebastian Potthoff produced the initial manuscript draft. All co‐authors provided input into drafts, and read and approved the final version of the manuscript.

## IMPLEMENTALL CONSORTIUM

Adriaan Hoogendoorn, Ainslie O'Connor, Alexis Whitton, Alison Calear, Andia Meksi, Anna Sofie Rømer, Anne Etzelmüller, Antoine Yrondi, Arlinda Cerga‐Pashoja, Besnik Loshaj, Bridianne O'Dea, Bruno Aouizerate, Camilla Stryhn, Carl May, Carmen Ceinos, Caroline Oehler, Catherine Pope, Christiaan Vis, Christine Marking, Claire R. van Genugten, Claus Duedal Pedersen, Corinna Gumbmann, Dana Menist, David Daniel Ebert, Denise Hanssen, Elena Heber, Els Dozeman, Emilie Brysting, Emmanuel Haffen, Enrico Zanalda, Erida Nelaj, Erik Van der Eycken, Eva Fris, Fiona Shand, Gentiana Qirjako, Géraldine Visentin, Heleen Riper, Helen Christensen, Ingrid Titzler, Isabel Weber, Isabel Zbukvic, Jeroen Ruwaard, Jerome Holtzmann, Johanna Freund, Johannes H Smit, Jordi Piera‐Jiménez, Josep Penya, Josephine Kreutzer, Josien Schuurmans, Judith Rosmalen, Juliane Hug, Kim Mathiasen, Kristian Kidholm, Kristine Tarp, Leah Bührmann, Linda Lisberg, Ludovic Samalin, Maite Arrillaga, Margot Fleuren, Maria Chovet, Marion Leboyer, Mette Atipei Craggs, Mette Maria Skjøth, Naim Fanaj, Nicole Cockayne, Philip J. Batterham, Pia Driessen, Pierre Michel Llorca, Rhonda Wilson, Ricardo Araya, Robin Kok, Sebastian Potthoff, Sergi García Redondo, Sevim Mustafa, Søren Lange Nielsen, Tim Rapley, Tracy Finch, Ulrich Hegerl, Virginie Tsilibaris, Wissam Elhage and Ylenia Sacco.

## CONFLICT OF INTEREST STATEMENT

The authors declare no conflict of interest.

## ETHICS STATEMENT

Ethical approval for the process evaluation was granted by the University of Northumbria, UK (Submission Ref: 11039). Participants were required to provide and sign a written informed consent indicating the purpose of the study, the nature, use and management of their data.

## Supporting information

Supporting information.

Supporting information.

Supporting information.

Supporting information.

Supporting information.

## Data Availability

The data that support the findings of this study are available on request from the corresponding author. The data are not publicly available due to privacy or ethical restrictions. The data sets generated and/or analysed during the current study are not publicly available because this would likely compromise participants' anonymity. Some descriptive data may be available from the corresponding author upon reasonable request.
