# Supplementary material for: Towards an Implementation‐STakeholder Engagement Model (I‐STEM) for improving health and social care services
Source: Health Expect. 2023 Jul 4;26(5):1997–2012. doi: 10.1111/hex.13808 (PMC10485327; doi:10.1111/hex.13808)
Supplement: Supplementary file 1 — Supporting information. [file HEX-26--s002.docx]

**Additional File 1**

*ImpleMentAll Process Evaluation Protocol*

Contents

[1. Background 2](#_Toc134787819)

[1.1. Introduction 2](#_Toc134787820)

[1.2. ItFits-toolkit 2](#_Toc134787821)

[1.3. Implementation of ItFits-toolkit across IMA sites 2](#_Toc134787822)

[2. Methods 3](#_Toc134787823)

[2.1. Design 3](#_Toc134787824)

[2.2. Sample 3](#_Toc134787825)

[2.3. Recruitment 4](#_Toc134787826)

[2.4. Data collection 4](#_Toc134787827)

[2.4.1. Interviews 4](#_Toc134787828)

[2.4.2. Observations 5](#_Toc134787829)

[2.4.3. Data management and anonymisation 5](#_Toc134787830)

[2.5. Data coding and interpretation 5](#_Toc134787831)

[3. References 7](#_Toc134787832)

[4. Appendix 1: ItFits-Toolkit Process 8](#_Toc134787833)

[5. Appendix 1: Example Interview Topic Guide 9](#_Toc134787834)

# Background

## Introduction

ImpleMentAll (IMA) aims to examine the effectiveness of tailored implementation compared to usual implementation of Internet-based Cognitive Behavioural Therapy (iCBT) in routine practice, in relation to implementation outcomes. There are three key evaluation components of the IMA study: Effectiveness study; Implementation as Usual (IAU) study; and Process Evaluation study. This document provides a detailed protocol for the Process Evaluation study. More details about the effectiveness and IAU study can be found in the published trial protocol (Bührmann et al., 2020).

## ItFits-toolkit

In the IMA study, tailored implementation is operationalised in Integrated Theory-based Framework for Intervention Tailoring Strategies (the ItFits-toolkit; see Appendix 1). The ItFits-toolkit is based on concepts of implementation tailoring and is theoretically informed (Normalization Process Theory (NPT) and Evidence-based Quality Improvement). Through a digital platform, the ItFits-toolkit provides implementers with guidance, resources, and support to identify and address key barriers to iCBT implementation. It supports a team-based approach to developing and undertaking tailored implementation strategies, and consists of four key modules:

1) identifying and prioritising implementation goals and barriers to reaching these goals,

2) matching of implementation barriers to strategies,

3) designing a plan for carrying out strategies in a local context, and

4) applying strategies and reviewing progress.

The ItFits-toolkit is designed to work in a range of complex and diverse settings, organisations, and healthcare systems; and was developed to be structured but also flexible.

## Implementation of ItFits-toolkit across IMA sites

The ItFits-toolkit will be introduced to twelve implementation sites across nine countries (Europe and Australia) as a part of the IMA effectiveness study. In the trial, study sites will cross over from IAU to using the ItFits-toolkit, two sites at a time, at three monthly intervals. Sites will be required to work with the toolkit for at least six months, aiming to finish a complete cycle of the ItFits-toolkit process (all four modules) within this time to achieve an adequate exposure to the core working components of the toolkit.

A protocolised (online) introductory training on using the toolkit will be provided to implementation teams at each site. Periodic support will also be provided, including monthly teleconferences with sites during their active phase, solely focusing on technical questions about the toolkit itself and to ensure a smooth working process. A distinction will be made between technical support with using the online toolkit (which was provided), and potential questions and requests from sites relating to the implementation work itself (e.g., barriers identification or decisions about strategies to implement). Questions concerning the latter will not be addressed by the guidance team, as this will be part of the work that implementation teams will be required to complete independently using the ItFits-toolkit.

# Methods

## Design

An in-depth qualitative process evaluation will be conducted alongside the IMA effectiveness study and will focus on engagement, embedding and integration of the ItFits-toolkit by its users in their implementation work. Three questions will be explored:

1. How do IMA implementation teams engage with the ItFits-toolkit?
2. How is the ItFits-toolkit reconfigured and adapted within and across settings?
3. What factors shape implementation of the ItFits-toolkit?

The study design will consist of qualitative interviews with members of the implementation teams, observations of meetings and events related to their work with the toolkit. Ethical approval for the process evaluation was granted by the University of Northumbria, UK.

## Sample

Individual participants in the qualitative study will include members of the site implementation teams: Implementation Leads (ILs), core team members who will be part of the teams and working closely with ILs, and other individuals involved in the implementation work. These ‘other’ individuals will include stakeholders who have key roles in relation to the work undertaken through the ItFits-toolkit, but who are not considered part of the core implementation team, for example as key facilitators working in collaborating organisations that form part of service delivery. Such individuals will be identified on a site-by-site basis after interviews with implementation team members. IL and core team member participants will be professionals working within healthcare within both private and public sectors who are involved in the implementation of the iCBT service. This will include clinical staff, managers and policy makers. Data collection will not include patients or their data.

## Recruitment

Sites participating in the ImpleMentAll trial are already aware that they will be taking part in the process evaluation activities described in this application. In making individual approaches, we will contact the ILs within the sites soon after the site has been informed of their entry point (3 months ahead) into the active phase of the trial, to arrange an initial interview. From this initial contact, we will identify further team members to approach for interview and arrange to conduct multiple interviews within a site in a single visit or around a similar time-point (if not conducted in person). All participants will be provided with an information sheet and participants who will take part in the interviews will also receive a topic guide. Written consent will be obtained after a period of at least 24 hours for reflection. In addition, during the interviews the researcher will check if the participant wishes to continue with the interview and is happy at the end for the content to be used. For all the research activities we will undertake the same consent process.

Overview of consent procedures:

1. All potential participants will be informed of the study by the researcher.

2. All potential participants will receive a study information sheet.

3. At least 24 hours to consider decision.

4. Written consent obtained by researcher.

5. Participants interviewed/ observed.

In line with the GDPR the information sheet will provide participants with clear information about how their data will be processed. Furthermore, the information sheet will provide participants with their data subject rights, including their right to access all personal data held about them, their right to correct incomplete/inaccurate information, their right to request data processing to be ceased etc.

## Data collection

### Interviews

Semi-structured interviews with ILs, core team members and stakeholders within implementation sites will be conducted using a theory-informed topic guide (see Appendix 1) (May et al., 2009). An initial interview will be conducted with the IL at the study site shortly after the site begins the active part of the trial. The purpose of the interviews will be to explore how the team experience ‘implementation as usual’ prior to using the toolkit, what they perceive as the barriers to implementation, how they intend to address them, and their initial thoughts about the toolkit. Follow up interviews will be conducted with ILs in the middle of the active period (around 3-4 months after the start of their trial) and again after completing the active period (6-7 months after the start of their trial). Core team members and stakeholders will also be interviewed to gain in-depth data on specific issues. Interviews will be conducted remotely using teleconferencing technology, mostly on an individual basis, but some interviews may include multiple team members at once. All interviews will be conducted in English by SP, a post-doctoral researcher with extensive experience in conducting qualitative research.

### Observations

Meetings and events related to use of the ItFits-toolkit will be observed, either in real time or via recordings made by site and project team members. These include introductory sessions, monthly group support calls, follow-up calls at one month and at 6 months (after sites’ six month ‘active’ toolkit phase), ad-hoc on-demand technical support, closing sessions, and core team meetings. All support activities are to be delivered by the guidance team either face-to-face or via videoconferencing technology. To ensure clear distinction between guidance support and evaluation, a member of the process evaluation team (SP) will act as an observer only and will not be involved in support activities. The purpose of the observations will be to gain in-depth, real-world insights into how implementers work with the ItFits-toolkit to tailor implementation strategies.

### Data management and anonymisation

All data will be securely managed, with password protection. Interviews will be audio-recorded with participants’ consent and transcribed verbatim for analysis. Meetings and events will be observed and fieldnotes taken with participants’ consent. Some of those meetings and events will also be audio-recorded with participants’ consent. Only selected meetings and events, or moments within them, that were deemed especially rich in detail, will be transcribed. Fieldnotes will be electronically recorded as typed documents. All data will be carefully anonymised to prevent identification of either the individual participant or the participating study site. Anonymised data only will be shared with the wider process evaluation team (including the implementation-as-usual team) for analysis purposes. Qualitative software (NVivo/Dedoose) will be used to support the data management and analysis process and establish an audit trail.

## Data coding and interpretation

We will draw on the standard procedures of rigorous qualitative analysis, including pre-coding, open and focused coding, constant comparison, memoing, tables, diagrams, and deviant case analysis (Rapley, 2011). Analysis will occur concurrently with data collection following the stepped order of implementation sites’ cross-over to the ItFits-condition. This will allow for emerging ideas and concepts found in earlier rounds of fieldwork to be explored in subsequent ones. A team-based approach to data interpretation, coding, and analysis will ensure that a range of possible interpretations of data can be explored. Bespoke training in qualitative methodology will be provided to the research team by the work package leads, to establish a minimum level of understanding of qualitative analytical approaches across team members for participation in data analysis sessions. Regular ‘data clinics’ (joint analysis meetings) will be held where a section of transcribed data will be discussed and interpreted jointly by team members. Early analysis sessions will involve working with smaller sections of transcripts, noting and discussing issues of interest, and sharing different interpretations of data discussed. Over time, these meetings will progress towards the development of categorised data ‘codes’ and analytic concepts, which will be iteratively developed and refined through successive data clinics and through detailed application to the data set. Key analytical ideas about tailored implementation and use of the ItFits-toolkit will be developed through team-based analysis workshops, led by the process evaluation team. This process will involve moving from team-generated data coding, to developing higher order understandings of the processes being described through the data by exploring patterns and differences across and between study sites.

## Dissemination

Various channels will be used to disseminate the results of the IMA process evaluation. Firstly, the results will be presented to the IMA consortium at a final project conference. This conference will be attended by a diverse, multi-national group of stakeholders, including researchers, practitioners, policy makers, industry partners, and patient representatives. Project reports will be made openly accessible via the European Commission’s project repository. Later, results will be published in implementation related open-access, peer reviewed journals and presented at both national and international conferences.

# References

Bührmann, L., Schuurmans, J., Ruwaard, J., Fleuren, M., Etzelmüller, A., Piera-Jiménez, J., . . . on behalf of the ImpleMentAll, c. (2020). Tailored implementation of internet-based cognitive behavioural therapy in the multinational context of the ImpleMentAll project: a study protocol for a stepped wedge cluster randomized trial. *Trials*, *21*(1), 893. <https://doi.org/10.1186/s13063-020-04686-4>

May, C. R., Mair, F., Finch, T., MacFarlane, A., Dowrick, C., Treweek, S., . . . Rogers, A. (2009). Development of a theory of implementation and integration: Normalization Process Theory. *Implementation Science*, *4*(1), 29.

Rapley, T. (2011). Some pragmatics of data analysis. *Qualitative Research*, *3*, 273-290.

# **Appendix 1:** ItFits-Toolkit Process


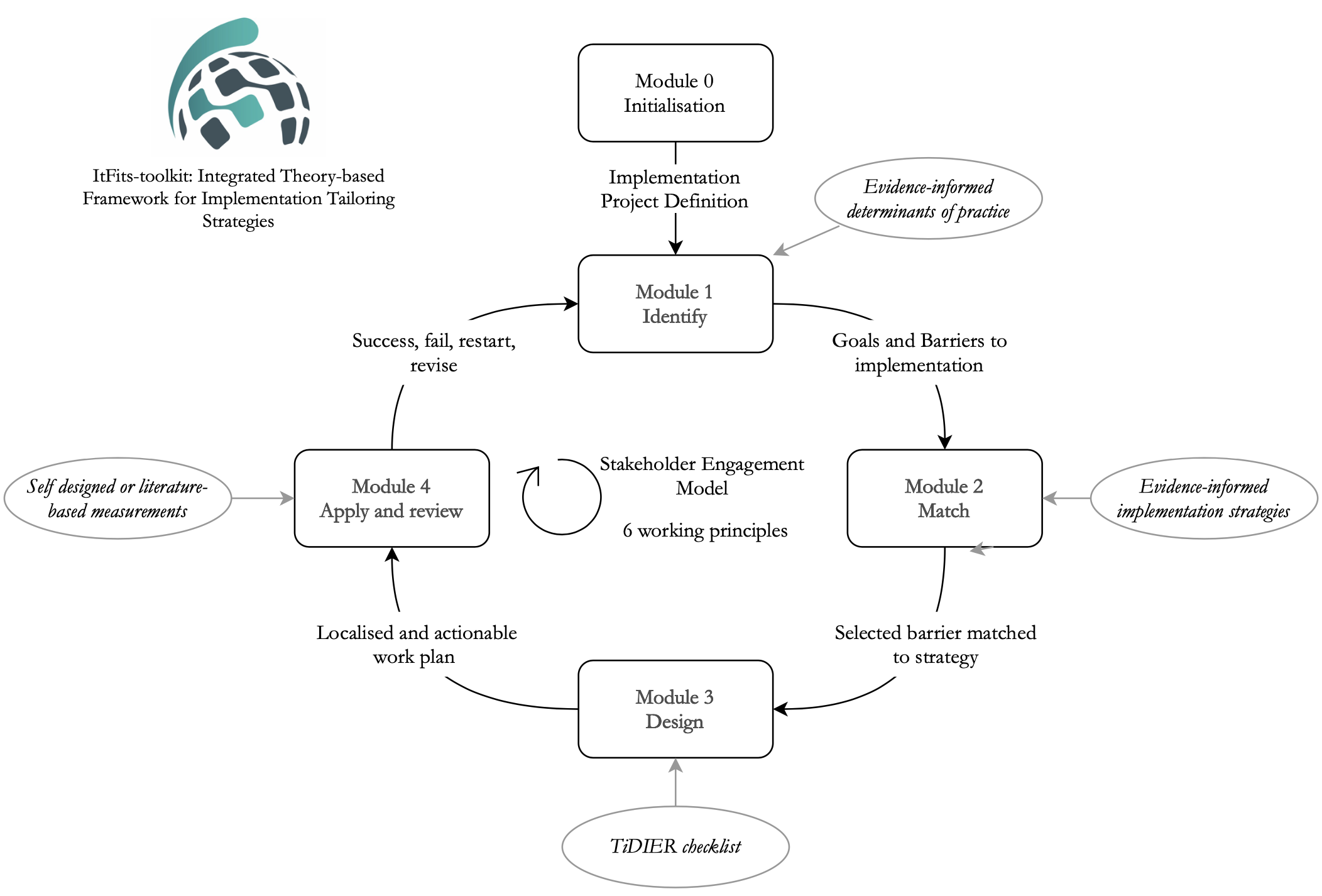


Module 1) identifying and prioritising implementation goals and barriers to reaching these goals, Module 2) matching barriers to implementation strategies, Module 3) designing a work plan for carrying out the strategies, and Module 4) applying strategies and reviewing progress.

# **Appendix 1:** Example Interview Topic Guide

*Note: The interview schedule is developmental and will change over time. The questions will need to be tailored to the specific answers of each interviewee. The questions will also be developed from the findings of prior interviews, as well as observations. The interview schedule given here is therefore a general topic guide for the qualitative interviews.*

**Introduction**

- Explain purpose of the study and this interview
- Explain interview recorded but details will be confidential
- Explore any questions or concerns?
- Re-affirm consent

**About your role**

Could you tell me about your role in the organization?

- Job title
- Responsibilities

**Clinical service**

Could you tell me a bit about the iCBT service you trying to implement? [*Compare to data we already have*]

- Evidence-based
- People involved
- Stage of implementation

**Implementation as usual**

Do you remember what you did to improve your service before receiving the ItFits-toolkit toolkit? [*Compare with data we already have*]

- Setting up the service for the trial
- Improving implementation of service
- Timing of effort
- Stakeholder engagement
- Barriers
- Successes

**Minimal guidance**

Could you tell me a bit about the guidance you received to support your use of the ItFits-toolkit toolkit?

- Introductory session
- Monthly group support calls
- Closing session
- Ad-hoc technical assistance

**Implementation with ItFits-toolkit**

Can you walk me through how you have been using the ItFits-toolkit toolkit so far?

- People involved (roles/ understanding/ commitment/ enrolment)
- Likes/ dislikes
- Changes in working approach
- Principles
- Implementation project(s)
- Goals and barriers (identification/ prioritization)
- Stakeholder engagement

**Follow-up interviews at T2 and T3** [*Note that this will depend on the progress that sites have made*]

- Strategies
- TIDieR
- Sub projects
- Plan to assess impact
- Monitor
- Review (reflections)

**Individual user experience of ItFits-toolkit**

Since you started using ItFits-toolkit, how many times have you used it?

- Motivations
- Expected outcomes
- Planning
- Barriers/ facilitators
- Coping planning
- Routines/ habit

**Additional File 2**

*Completed ‘Standards for Reporting Qualitative Research’ (SRQR) Checklist.*

|  | **Standards for Reporting Qualitative Research (SRQR)** |  |
| --- | --- | --- |
|  | <http://www.equator-network.org/reporting-guidelines/srqr/>  O'Brien BC, Harris IB, Beckman TJ, Reed DA, Cook DA. Standards for reporting qualitative research: a synthesis of recommendations. Acad Med. 2014 Sep;89(9):1245-51. doi: 10.1097/ACM.0000000000000388. PMID: 24979285. | |
|  |  |  |
| **Title and abstract** | | **Page numbers** |
|  | **Title** - Concise description of the nature and topic of the study Identifying the study as qualitative or indicating the approach (e.g., ethnography, grounded theory) or data collection methods (e.g., interview, focus group) is recommended | Title page |
|  | **Abstract** - Summary of key elements of the study using the abstract format of the intended publication; typically includes background, purpose, methods, results, and conclusions | 1-2 |
| **Introduction** | |  |
|  | **Problem formulation** - Description and significance of the problem/phenomenon studied; review of relevant theory and empirical work; problem statement | 3-6 |
|  | **Purpose or research question** - Purpose of the study and specific objectives or questions | 6 |
| **Methods** | |  |
|  | **Qualitative approach and research paradigm** - Qualitative approach (e.g., ethnography, grounded theory, case study, phenomenology, narrative research) and guiding theory if appropriate; identifying the research paradigm (e.g., postpositivist, constructivist/ interpretivist) is also recommended; rationale** | 6-10 |
|  | **Researcher characteristics and reflexivity** - Researchers’ characteristics that may influence the research, including personal attributes, qualifications/experience, relationship with participants, assumptions, and/or presuppositions; potential or actual interaction between researchers’ characteristics and the research questions, approach, methods, results, and/or transferability | 6-10 |
|  | **Context** - Setting/site and salient contextual factors; rationale | 6-9 |
|  | **Sampling strategy** - How and why research participants, documents, or events were selected; criteria for deciding when no further sampling was necessary (e.g., sampling saturation); rationale | 9 |
|  | **Ethical issues pertaining to human subjects** - Documentation of approval by an appropriate ethics review board and participant consent, or explanation for lack thereof; other confidentiality and data security issues | Title page |
|  | **Data collection methods** - Types of data collected; details of data collection procedures including (as appropriate) start and stop dates of data collection and analysis, iterative process, triangulation of sources/methods, and modification of procedures in response to evolving study findings; rationale | 9 |
|  | **Data collection instruments and technologies** - Description of instruments (e.g., interview guides, questionnaires) and devices (e.g., audio recorders) used for data collection; if/how the instrument(s) changed over the course of the study | 8-9 |
|  | **Units of study** - Number and relevant characteristics of participants, documents, or events included in the study; level of participation (could be reported in results) | 9 |
|  | **Data processing** - Methods for processing data prior to and during analysis, including transcription, data entry, data management and security, verification of data integrity, data coding, and anonymization/de-identification of excerpts | 9 |
|  | **Data analysis** - Process by which inferences, themes, etc., were identified and developed, including the researchers involved in data analysis; usually references a specific paradigm or approach; rationale | 10 |
|  | **Techniques to enhance trustworthiness** - Techniques to enhance trustworthiness and credibility of data analysis (e.g., member checking, audit trail, triangulation); rationale | 10 |
| **Results/findings** | |  |
|  | **Synthesis and interpretation** - Main findings (e.g., interpretations, inferences, and themes); might include development of a theory or model, or integration with prior research or theory | 11-17 |
|  | **Links to empirical data** - Evidence (e.g., quotes, field notes, text excerpts, photographs) to substantiate analytic findings | 30-39 |
| **Discussion** | |  |
|  | **Integration with prior work, implications, transferability, and contribution(s) to the field -** Short summary of main findings; explanation of how findings and conclusions connect to, support, elaborate on, or challenge conclusions of earlier scholarship; discussion of scope of application/generalizability; identification of unique contribution(s) to scholarship in a discipline or field | 17-22 |
|  | **Limitations** - Trustworthiness and limitations of findings | 21-22 |
| **Other** | |  |
|  | **Conflicts of interest** - Potential sources of influence or perceived influence on study conduct and conclusions; how these were managed | Title page |
|  | **Funding** - Sources of funding and other support; role of funders in data collection, interpretation, and reporting | Title page |

**Reference:**

O'Brien BC, Harris IB, Beckman TJ, Reed DA, Cook DA. Standards for reporting qualitative research: a synthesis of recommendations. Acad Med. 2014 Sep;89(9):1245-51. doi: 10.1097/ACM.0000000000000388. PMID: 24979285.

**Additional File 3**

*Two early iterations of the Implementation-STakeholder Engagement Model (I-STEM)*

Version 1

Version 2

**Additional File 4**

*Applied case example* *of the Implementation-Stakeholder Engagement Model (I-STEM)*

The following case example from the IMA process evaluation demonstrates the workability of the I-STEM. Implementers in the case study had two primary objectives for engaging stakeholders in the implementation work. Firstly, they wanted to get a better *understanding* of stakeholders’ views on ‘what are the barriers and what already works good’ [Site 1, interview 4] in terms of implementing the service. Secondly, implementers wanted stakeholders to help them *do* the implementation by ‘exploring new ways of recruitment’ [Site 1, interview 6] into the service. To achieve their objectives, implementers chose to engage with practitioners because of their *expertise* and *impact* on implementation of the service. For example, one of the stakeholders was ‘working as an eCoach for a long time’ and therefore, ‘had some kind of experience with the service delivery’ [Site 1, interview 2]. They also engaged with an external partner because of their positive *orientation* towards the service and their ability to reach new service users. Implementers at this site made use of a range of engagement approaches. They used surveys and focus groups to *assess* stakeholders’ views on barriers and facilitators. They also *collaborated* with stakeholders to co-produce a resource advertising the service to users and to help them register to the digital platform. Implementers described the importance of communicating *regularly* (‘more than once a week’ [Site 1, interview 3]) with stakeholders. Implementers and stakeholders divided up implementation related tasks so ‘it became really clear who was responsible (*accountable*) for what step’ [Site 1, interview 3]. The outcome of the engagement activities included a better *understanding* of the problem, better *access* to resources, and increased buy in from key stakeholders. They managed to *enrol* stakeholders in the implementation process by involving them in the core implementation team. They managed to *access* further resources to produce a more professional version of their initial resource. Importantly, engaging with stakeholders provided them with a more comprehensive *understanding* of barriers to implementation: ‘Field workers didn’t know which programme is the best for this specific person. So yeah, they said it could be helpful to provide access to these programmes for field workers so they can have a look at it and so they get a better idea of what these programmes provide’ [Site 1, interview 6].
