# Supplementary material for: Towards an Implementation‐STakeholder Engagement Model (I‐STEM) for improving health and social care services
Source: Health Expect. 2023 Jul 4;26(5):1997–2012. doi: 10.1111/hex.13808 (PMC10485327; doi:10.1111/hex.13808)
Supplement: Supplementary file 4 — Supporting information. [file HEX-26--s005.docx]

**Additional File 3**

*Two early iterations of the Implementation-STakeholder Engagement Model (I-STEM)*

Version 1

Version 2
