# Supplementary material for: Towards an Implementation‐STakeholder Engagement Model (I‐STEM) for improving health and social care services
Source: Health Expect. 2023 Jul 4;26(5):1997–2012. doi: 10.1111/hex.13808 (PMC10485327; doi:10.1111/hex.13808)
Supplement: Supplementary file 5 — Supporting information. [file HEX-26--s001.docx]

**Additional File 4**

*Applied case example* *of the Implementation-Stakeholder Engagement Model (I-STEM)*

The following case example from the IMA process evaluation demonstrates the workability of the I-STEM. Implementers in the case study had two primary objectives for engaging stakeholders in the implementation work. Firstly, they wanted to get a better *understanding* of stakeholders’ views on ‘what are the barriers and what already works good’ [Site 1, interview 4] in terms of implementing the service. Secondly, implementers wanted stakeholders to help them *do* the implementation by ‘exploring new ways of recruitment’ [Site 1, interview 6] into the service. To achieve their objectives, implementers chose to engage with practitioners because of their *expertise* and *impact* on implementation of the service. For example, one of the stakeholders was ‘working as an eCoach for a long time’ and therefore, ‘had some kind of experience with the service delivery’ [Site 1, interview 2]. They also engaged with an external partner because of their positive *orientation* towards the service and their ability to reach new service users. Implementers at this site made use of a range of engagement approaches. They used surveys and focus groups to *assess* stakeholders’ views on barriers and facilitators. They also *collaborated* with stakeholders to co-produce a resource advertising the service to users and to help them register to the digital platform. Implementers described the importance of communicating *regularly* (‘more than once a week’ [Site 1, interview 3]) with stakeholders. Implementers and stakeholders divided up implementation related tasks so ‘it became really clear who was responsible (*accountable*) for what step’ [Site 1, interview 3]. The outcome of the engagement activities included a better *understanding* of the problem, better *access* to resources, and increased buy in from key stakeholders. They managed to *enrol* stakeholders in the implementation process by involving them in the core implementation team. They managed to *access* further resources to produce a more professional version of their initial resource. Importantly, engaging with stakeholders provided them with a more comprehensive *understanding* of barriers to implementation: ‘Field workers didn’t know which programme is the best for this specific person. So yeah, they said it could be helpful to provide access to these programmes for field workers so they can have a look at it and so they get a better idea of what these programmes provide’ [Site 1, interview 6].
